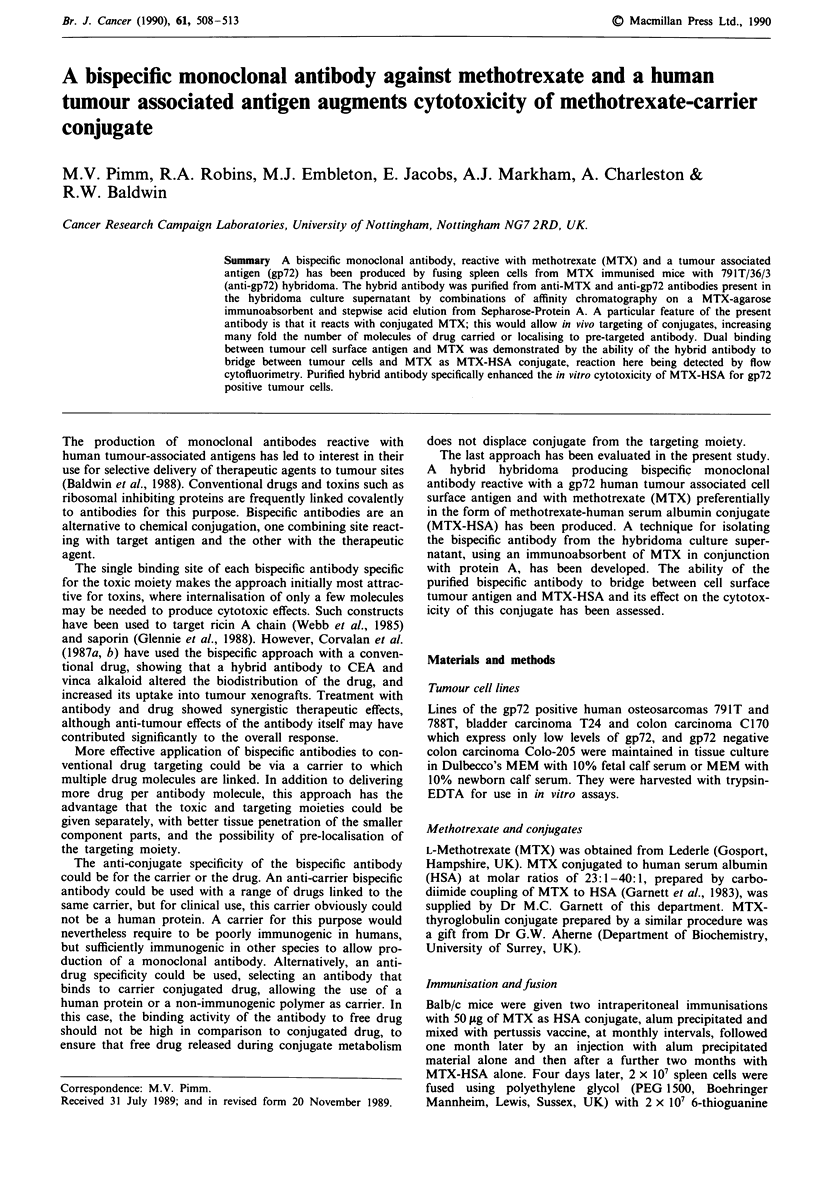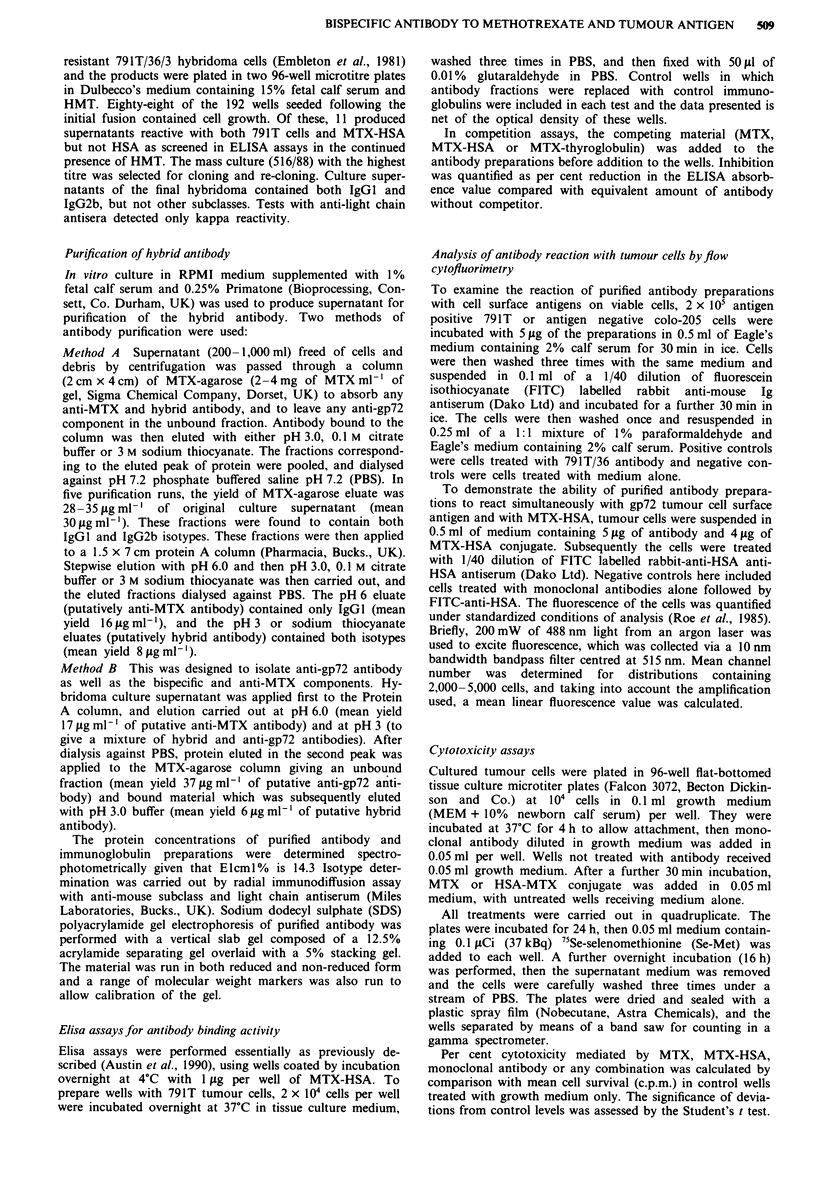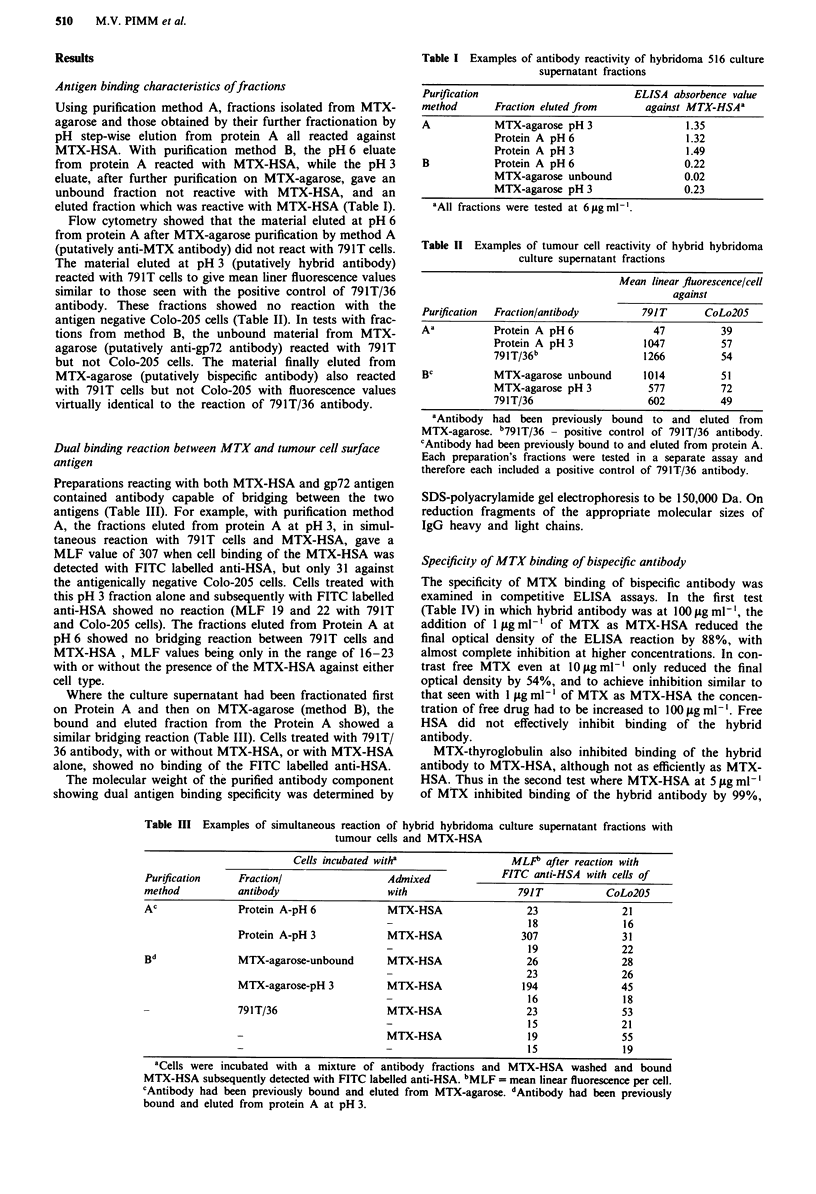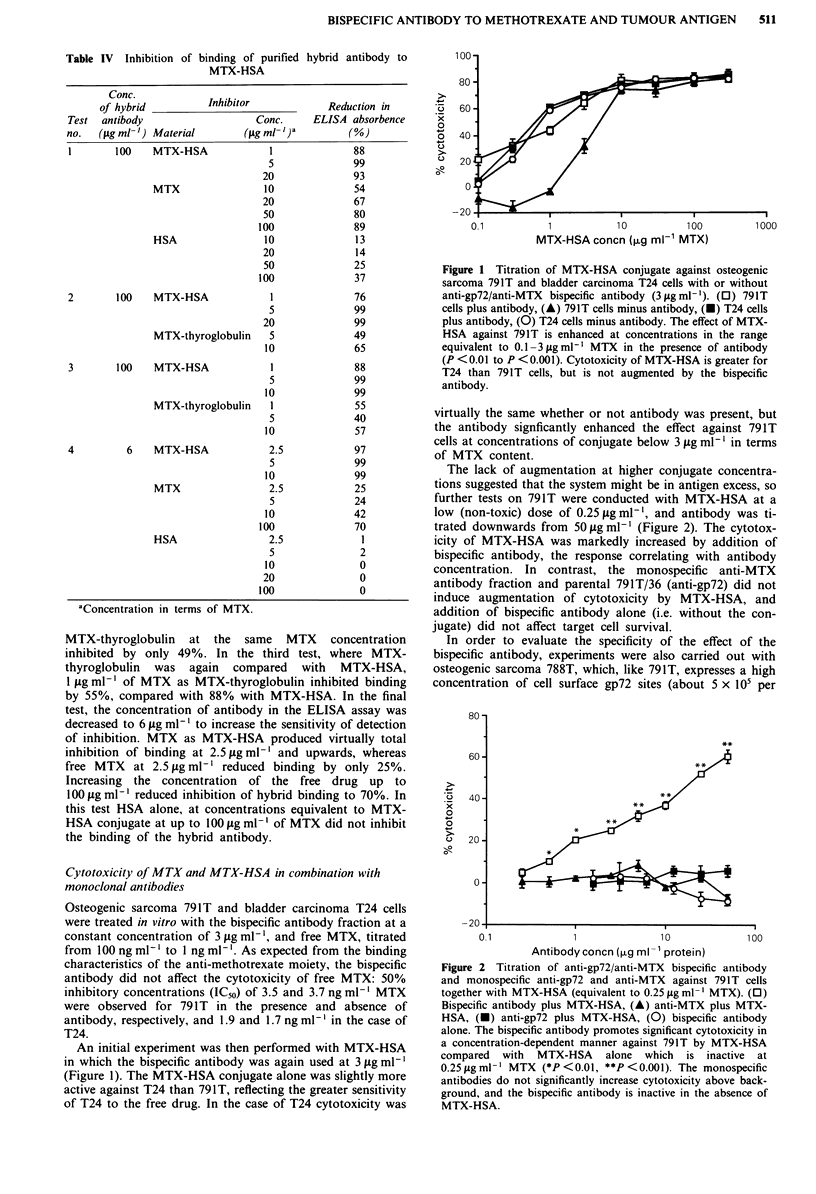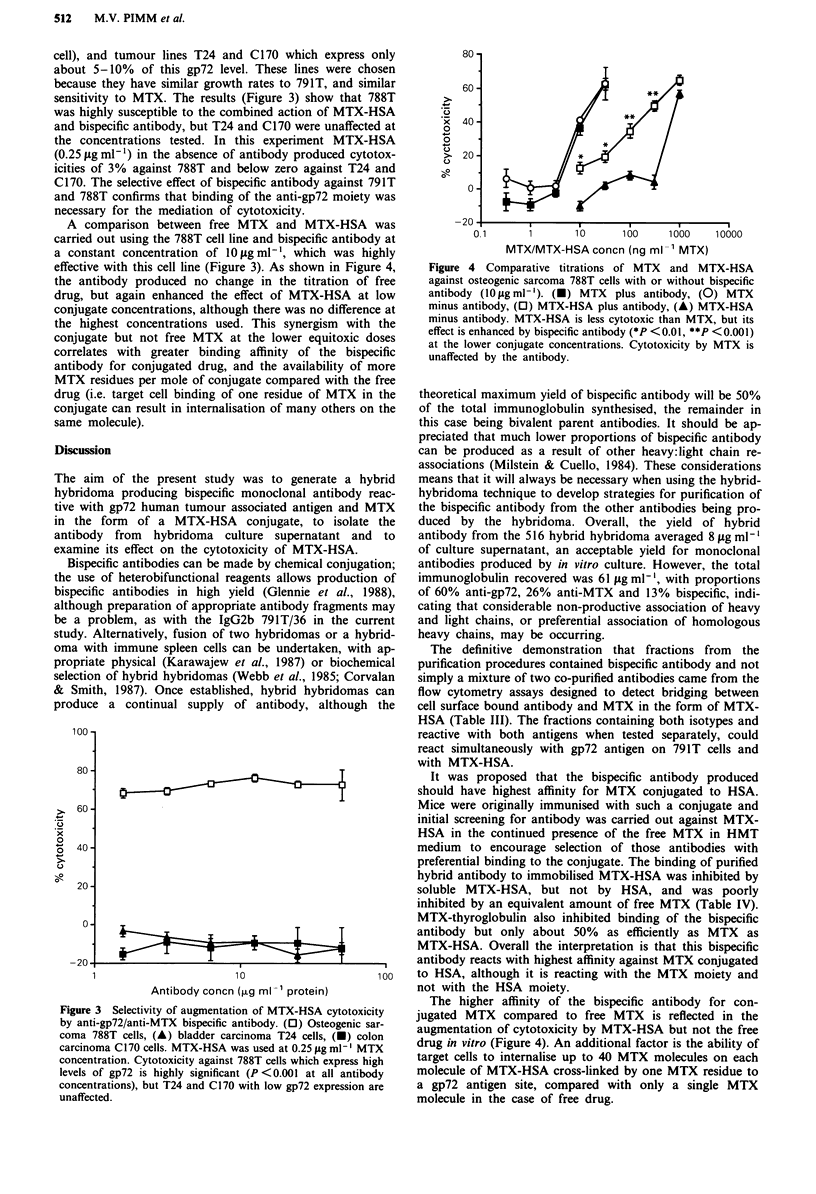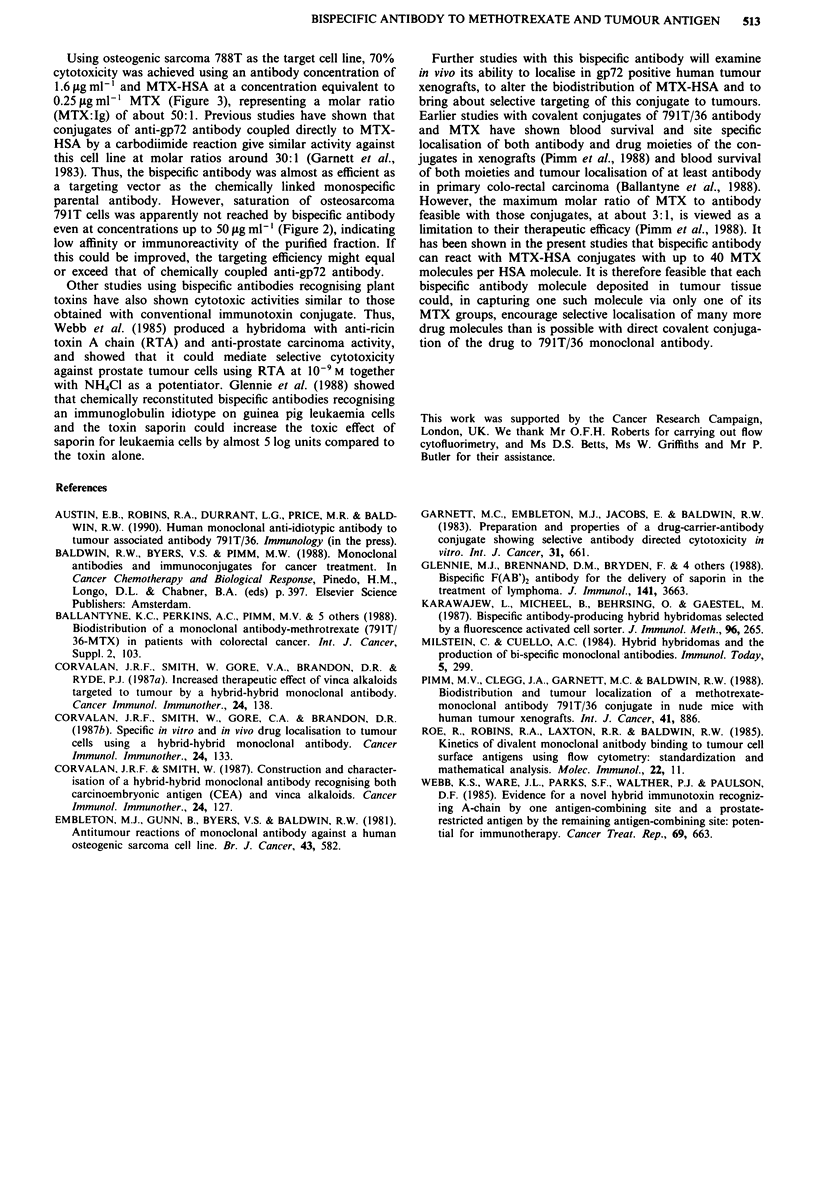# A bispecific monoclonal antibody against methotrexate and a human tumour associated antigen augments cytotoxicity of methotrexate-carrier conjugate.

**DOI:** 10.1038/bjc.1990.115

**Published:** 1990-04

**Authors:** M. V. Pimm, R. A. Robins, M. J. Embleton, E. Jacobs, A. J. Markham, A. Charleston, R. W. Baldwin

**Affiliations:** Cancer Research Campaign Laboratories, University of Nottingham, UK.

## Abstract

A bispecific monoclonal antibody, reactive with methotrexate (MTX) and a tumour associated antigen (gp72) has been produced by fusing spleen cells from MTX immunised mice with 791T/36/3 (anti-gp72) hybridoma. The hybrid antibody was purified from anti-MTX and anti-gp72 antibodies present in the hybridoma culture supernatant by combinations of affinity chromatography on a MTX-agarose immunoabsorbent and stepwise acid elution from Sepharose-Protein A. A particular feature of the present antibody is that it reacts with conjugated MTX; this would allow in vivo targeting of conjugates, increasing many fold the number of molecules of drug carried or localising to pre-targeted antibody. Dual binding between tumour cell surface antigen and MTX was demonstrated by the ability of the hybrid antibody to bridge between tumour cells and MTX as MTX-HSA conjugate, reaction here being detected by flow cytofluorimetry. Purified hybrid antibody specifically enhanced the in vitro cytotoxicity of MTX-HSA for gp72 positive tumour cells.


 © Macmillan Press Ltd., 1990

### A bispecific monoclonal antibody against methotrexate and a human tumour associated antigen augments cytotoxicity of methotrexate-carrier conjugate

M.V. Pimm, R.A. Robins, M.J. Embleton, E. Jacobs, A.J. Markham, A. Charleston & R.W. Baldwin

*Cancer Research Campaign Laboratories, University of Nottingham, Nottingham NG7 2RD, UK.*

**Summary** A bispecific monoclonal antibody, reactive with methotrexate (MTX) and a tumour associated antigen (gp72) has been produced by fusing spleen cells from MTX immunised mice with 791T/36/3 (anti-gp72) hybridoma. The hybrid antibody was purified from anti-MTX and anti-gp72 antibodies present in the hybridoma culture supernatant by combinations of affinity chromatography on a MTX-agarose immunoabsorbent and stepwise acid elution from Sepharose-Protein A. A particular feature of the present antibody is that it reacts with conjugated MTX; this would allow *in vivo* targeting of conjugates, increasing many fold the number of molecules of drug carried or localising to pre-targeted antibody. Dual binding between tumour cell surface antigen and MTX was demonstrated by the ability of the hybrid antibody to bridge between tumour cells and MTX as MTX-HSA conjugate, reaction here being detected by flow cytofluorimetry. Purified hybrid antibody specifically enhanced the *in vitro* cytotoxicity of MTX-HSA for gp72 positive tumour cells.

The production of monoclonal antibodes reactive with human tumour-associated antigens has led to interest in their use for selective delivery of therapeutic agents to tumour sites (Baldwin *et al.*, 1988). Conventional drugs and toxins such as ribosomal inhibiting proteins are frequently linked covalently to antibodies for this purpose. Bispecific antibodies are an alternative to chemical conjugation, one combining site reacting with target antigen and the other with the therapeutic agent.

The single binding site of each bispecific antibody specific for the toxic moiety makes the approach initially most attractive for toxins, where internalisation of only a few molecules may be needed to produce cytotoxic effects. Such constructs have been used to target ricin A chain (Webb *et al.*, 1985) and saporin (Glennie *et al.*, 1988). However, Corvalan *et al.* (1987*a*, *b*) have used the bispecific approach with a conventional drug, showing that a hybrid antibody to CEA and vinca alkaloid altered the biodistribution of the drug, and increased its uptake into tumour xenografts. Treatment with antibody and drug showed synergistic therapeutic effects, although anti-tumour effects of the antibody itself may have contributed significantly to the overall response.

More effective application of bispecific antibodies to conventional drug targeting could be via a carrier to which multiple drug molecules are linked. In addition to delivering more drug per antibody molecule, this approach has the advantage that the toxic and targeting moieties could be given separately, with better tissue penetration of the smaller component parts, and the possibility of pre-localisation of the targeting moiety.

The anti-conjugate specificity of the bispecific antibody could be for the carrier or the drug. An anti-carrier bispecific antibody could be used with a range of drugs linked to the same carrier, but for clinical use, this carrier obviously could not be a human protein. A carrier for this purpose would nevertheless require to be poorly immunogenic in humans, but sufficiently immunogenic in other species to allow production of a monoclonal antibody. Alternatively, an anti-drug specificity could be used, selecting an antibody that binds to carrier conjugated drug, allowing the use of a human protein or a non-immunogenic polymer as carrier. In this case, the binding activity of the antibody to free drug should not be high in comparison to conjugated drug, to ensure that free drug released during conjugate metabolism does not displace conjugate from the targeting moiety.

The last approach has been evaluated in the present study. A hybrid hybridoma producing bispecific monoclonal antibody reactive with a gp72 human tumour associated cell surface antigen and with methotrexate (MTX) preferentially in the form of methotrexate-human serum albumin conjugate (MTX-HSA) has been produced. A technique for isolating the bispecific antibody from the hybridoma culture supernatant, using an immunoabsorbent of MTX in conjunction with protein A, has been developed. The ability of the purified bispecific antibody to bridge between cell surface tumour antigen and MTX-HSA and its effect on the cytotoxicity of this conjugate has been assessed.

### Materials and methods

### *Tumour cell lines*

Lines of the gp72 positive human osteosarcomas 791T and 788T, bladder carcinoma T24 and colon carcinoma C170 which express only low levels of gp72, and gp72 negative colon carcinoma Colo-205 were maintained in tissue culture in Dulbecco's MEM with 10% fetal calf serum or MEM with 10% newborn calf serum. They were harvested with trypsin-EDTA for use in *in vitro* assays.

### *Methotrexate and conjugates*

L-Methotrexate (MTX) was obtained from Lederle (Gosport, Hampshire, UK). MTX conjugated to human serum albumin (HSA) at molar ratios of 23:1-40:1, prepared by carbodiimide coupling of MTX to HSA (Garnett *et al.*, 1983), was supplied by Dr M.C. Garnett of this department. MTX-thyroglobulin conjugate prepared by a similar procedure was a gift from Dr G.W. Aherne (Department of Biochemistry, University of Surrey, UK).

### *Immunisation and fusion*

Balb/c mice were given two intraperitoneal immunisations with 50 μg of MTX as HSA conjugate, alum precipitated and mixed with pertussis vaccine, at monthly intervals, followed one month later by an injection with alum precipitated material alone and then after a further two months with MTX-HSA alone. Four days later, $2 \times 10^7$ spleen cells were fused using polyethylene glycol (PEG 1500, Boehringer Mannheim, Lewis, Sussex, UK) with $2 \times 10^7$ 6-thioguanine

Correspondence: M.V. Pimm.
Received 31 July 1989; and in revised form 20 November 1989.

resistant 791T/36/3 hybridoma cells (Embleton *et al.*, 1981) and the products were plated in two 96-well microtitre plates in Dulbecco's medium containing 15% fetal calf serum and HMT. Eighty-eight of the 192 wells seeded following the initial fusion contained cell growth. Of these, 11 produced supernatants reactive with both 791T cells and MTX-HSA but not HSA as screened in ELISA assays in the continued presence of HMT. The mass culture (516/88) with the highest titre was selected for cloning and re-cloning. Culture supernatants of the final hybridoma contained both IgG1 and IgG2b, but not other subclasses. Tests with anti-light chain antisera detected only kappa reactivity.

*Purification of hybrid antibody*

*In vitro* culture in RPMI medium supplemented with 1% fetal calf serum and 0.25% Primatone (Bioprocessing, Consett, Co. Durham, UK) was used to produce supernatant for purification of the hybrid antibody. Two methods of antibody purification were used:

*Method A* Supernatant (200–1,000 ml) freed of cells and debris by centrifugation was passed through a column (2 cm × 4 cm) of MTX-agarose (2–4 mg of MTX $ml^{-1}$ of gel, Sigma Chemical Company, Dorset, UK) to absorb any anti-MTX and hybrid antibody, and to leave any anti-gp72 component in the unbound fraction. Antibody bound to the column was then eluted with either pH 3.0, 0.1 M citrate buffer or 3 M sodium thiocyanate. The fractions corresponding to the eluted peak of protein were pooled, and dialysed against pH 7.2 phosphate buffered saline pH 7.2 (PBS). In five purification runs, the yield of MTX-agarose eluate was 28–35 $\mu g\ ml^{-1}$ of original culture supernatant (mean 30 $\mu g\ ml^{-1}$). These fractions were found to contain both IgG1 and IgG2b isotypes. These fractions were then applied to a 1.5 × 7 cm protein A column (Pharmacia, Bucks., UK). Stepwise elution with pH 6.0 and then pH 3.0, 0.1 M citrate buffer or 3 M sodium thiocyanate was then carried out, and the eluted fractions dialysed against PBS. The pH 6 eluate (putatively anti-MTX antibody) contained only IgG1 (mean yield 16 $\mu g\ ml^{-1}$), and the pH 3 or sodium thiocyanate eluates (putatively hybrid antibody) contained both isotypes (mean yield 8 $\mu g\ ml^{-1}$).

*Method B* This was designed to isolate anti-gp72 antibody as well as the bispecific and anti-MTX components. Hybridoma culture supernatant was applied first to the Protein A column, and elution carried out at pH 6.0 (mean yield 17 $\mu g\ ml^{-1}$ of putative anti-MTX antibody) and at pH 3 (to give a mixture of hybrid and anti-gp72 antibodies). After dialysis against PBS, protein eluted in the second peak was applied to the MTX-agarose column giving an unbound fraction (mean yield 37 $\mu g\ ml^{-1}$ of putative anti-gp72 antibody) and bound material which was subsequently eluted with pH 3.0 buffer (mean yield 6 $\mu g\ ml^{-1}$ of putative hybrid antibody).

The protein concentrations of purified antibody and immunoglobulin preparations were determined spectrophotometrically given that E1cm1% is 14.3 Isotype determination was carried out by radial immunodiffusion assay with anti-mouse subclass and light chain antiserum (Miles Laboratories, Bucks., UK). Sodium dodecyl sulphate (SDS) polyacrylamide gel electrophoresis of purified antibody was performed with a vertical slab gel composed of a 12.5% acrylamide separating gel overlaid with a 5% stacking gel. The material was run in both reduced and non-reduced form and a range of molecular weight markers was also run to allow calibration of the gel.

*Elisa assays for antibody binding activity*

Elisa assays were performed essentially as previously described (Austin *et al.*, 1990), using wells coated by incubation overnight at 4°C with 1 $\mu g$ per well of MTX-HSA. To prepare wells with 791T tumour cells, $2 \times 10^4$ cells per well were incubated overnight at 37°C in tissue culture medium, washed three times in PBS, and then fixed with 50 $\mu l$ of 0.01% glutaraldehyde in PBS. Control wells in which antibody fractions were replaced with control immunoglobulins were included in each test and the data presented is net of the optical density of these wells.

In competition assays, the competing material (MTX, MTX-HSA or MTX-thyroglobulin) was added to the antibody preparations before addition to the wells. Inhibition was quantified as per cent reduction in the ELISA absorbence value compared with equivalent amount of antibody without competitor.

*Analysis of antibody reaction with tumour cells by flow cytofluorimetry*

To examine the reaction of purified antibody preparations with cell surface antigens on viable cells, $2 \times 10^5$ antigen positive 791T or antigen negative colo-205 cells were incubated with 5 $\mu g$ of the preparations in 0.5 ml of Eagle's medium containing 2% calf serum for 30 min in ice. Cells were then washed three times with the same medium and suspended in 0.1 ml of a 1/40 dilution of fluorescein isothiocyanate (FITC) labelled rabbit anti-mouse Ig antiserum (Dako Ltd) and incubated for a further 30 min in ice. The cells were then washed once and resuspended in 0.25 ml of a 1:1 mixture of 1% paraformaldehyde and Eagle's medium containing 2% calf serum. Positive controls were cells treated with 791T/36 antibody and negative controls were cells treated with medium alone.

To demonstrate the ability of purified antibody preparations to react simultaneously with gp72 tumour cell surface antigen and with MTX-HSA, tumour cells were suspended in 0.5 ml of medium containing 5 $\mu g$ of antibody and 4 $\mu g$ of MTX-HSA conjugate. Subsequently the cells were treated with 1/40 dilution of FITC labelled rabbit-anti-HSA anti-HSA antiserum (Dako Ltd). Negative controls here included cells treated with monoclonal antibodies alone followed by FITC-anti-HSA. The fluorescence of the cells was quantified under standardized conditions of analysis (Roe *et al.*, 1985). Briefly, 200 mW of 488 nm light from an argon laser was used to excite fluorescence, which was collected via a 10 nm bandwidth bandpass filter centred at 515 nm. Mean channel number was determined for distributions containing 2,000–5,000 cells, and taking into account the amplification used, a mean linear fluorescence value was calculated.

*Cytotoxicity assays*

Cultured tumour cells were plated in 96-well flat-bottomed tissue culture microtiter plates (Falcon 3072, Becton Dickinson and Co.) at $10^4$ cells in 0.1 ml growth medium (MEM + 10% newborn calf serum) per well. They were incubated at 37°C for 4 h to allow attachment, then monoclonal antibody diluted in growth medium was added in 0.05 ml per well. Wells not treated with antibody received 0.05 ml growth medium. After a further 30 min incubation, MTX or HSA-MTX conjugate was added in 0.05 ml medium, with untreated wells receiving medium alone.

All treatments were carried out in quadruplicate. The plates were incubated for 24 h, then 0.05 ml medium containing 0.1 $\mu Ci$ (37 kBq) $^{75}$Se-selenomethionine (Se-Met) was added to each well. A further overnight incubation (16 h) was performed, then the supernatant medium was removed and the cells were carefully washed three times under a stream of PBS. The plates were dried and sealed with a plastic spray film (Nobecutane, Astra Chemicals), and the wells separated by means of a band saw for counting in a gamma spectrometer.

Per cent cytotoxicity mediated by MTX, MTX-HSA, monoclonal antibody or any combination was calculated by comparison with mean cell survival (c.p.m.) in control wells treated with growth medium only. The significance of deviations from control levels was assessed by the Student's *t* test.

### Results

### Antigen binding characteristics of fractions

Using purification method A, fractions isolated from MTX-agarose and those obtained by their further fractionation by pH step-wise elution from protein A all reacted against MTX-HSA. With purification method B, the pH 6 eluate from protein A reacted with MTX-HSA, while the pH 3 eluate, after further purification on MTX-agarose, gave an unbound fraction not reactive with MTX-HSA, and an eluted fraction which was reactive with MTX-HSA (Table I).

Flow cytometry showed that the material eluted at pH 6 from protein A after MTX-agarose purification by method A (putatively anti-MTX antibody) did not react with 791T cells. The material eluted at pH 3 (putatively hybrid antibody) reacted with 791T cells to give mean liner fluorescence values similar to those seen with the positive control of 791T/36 antibody. These fractions showed no reaction with the antigen negative Colo-205 cells (Table II). In tests with fractions from method B, the unbound material from MTX-agarose (putatively anti-gp72 antibody) reacted with 791T but not Colo-205 cells. The material finally eluted from MTX-agarose (putatively bispecific antibody) also reacted with 791T cells but not Colo-205 with fluorescence values virtually identical to the reaction of 791T/36 antibody.

### Dual binding reaction between MTX and tumour cell surface antigen

Preparations reacting with both MTX-HSA and gp72 antigen contained antibody capable of bridging between the two antigens (Table III). For example, with purification method A, the fractions eluted from protein A at pH 3, in simultaneous reaction with 791T cells and MTX-HSA, gave a MLF value of 307 when cell binding of the MTX-HSA was detected with FITC labelled anti-HSA, but only 31 against the antigenically negative Colo-205 cells. Cells treated with this pH 3 fraction alone and subsequently with FITC labelled anti-HSA showed no reaction (MLF 19 and 22 with 791T and Colo-205 cells). The fractions eluted from Protein A at pH 6 showed no bridging reaction between 791T cells and MTX-HSA , MLF values being only in the range of 16–23 with or without the presence of the MTX-HSA against either cell type.

Where the culture supernatant had been fractionated first on Protein A and then on MTX-agarose (method B), the bound and eluted fraction from the Protein A showed a similar bridging reaction (Table III). Cells treated with 791T/36 antibody, with or without MTX-HSA, or with MTX-HSA alone, showed no binding of the FITC labelled anti-HSA.

The molecular weight of the purified antibody component showing dual antigen binding specificity was determined by SDS-polyacrylamide gel electrophoresis to be 150,000 Da. On reduction fragments of the appropriate molecular sizes of IgG heavy and light chains.

**Table I** Examples of antibody reactivity of hybridoma 516 culture supernatant fractions

| *Purification method* | *Fraction eluted from* | *ELISA absorbence value against MTX-HSA*[a] |
|---|---|---|
| A | MTX-agarose pH 3 | 1.35 |
| | Protein A pH 6 | 1.32 |
| | Protein A pH 3 | 1.49 |
| B | Protein A pH 6 | 0.22 |
| | MTX-agarose unbound | 0.02 |
| | MTX-agarose pH 3 | 0.23 |

[a]All fractions were tested at 6 μg ml$^{-1}$.

**Table II** Examples of tumour cell reactivity of hybrid hybridoma culture supernatant fractions

| | | *Mean linear fluorescence/cell against* | |
|---|---|---|---|
| *Purification* | *Fraction/antibody* | *791T* | *CoLo205* |
| A[a] | Protein A pH 6 | 47 | 39 |
| | Protein A pH 3 | 1047 | 57 |
| | 791T/36[b] | 1266 | 54 |
| B[c] | MTX-agarose unbound | 1014 | 51 |
| | MTX-agarose pH 3 | 577 | 72 |
| | 791T/36 | 602 | 49 |

[a]Antibody had been previously bound to and eluted from MTX-agarose. [b]791T/36 – positive control of 791T/36 antibody. [c]Antibody had been previously bound to and eluted from protein A. Each preparation's fractions were tested in a separate assay and therefore each included a positive control of 791T/36 antibody.

### Specificity of MTX binding of bispecific antibody

The specificity of MTX binding of bispecific antibody was examined in competitive ELISA assays. In the first test (Table IV) in which hybrid antibody was at 100 μg ml$^{-1}$, the addition of 1 μg ml$^{-1}$ of MTX as MTX-HSA reduced the final optical density of the ELISA reaction by 88%, with almost complete inhibition at higher concentrations. In contrast free MTX even at 10 μg ml$^{-1}$ only reduced the final optical density by 54%, and to achieve inhibition similar to that seen with 1 μg ml$^{-1}$ of MTX as MTX-HSA the concentration of free drug had to be increased to 100 μg ml$^{-1}$. Free HSA did not effectively inhibit binding of the hybrid antibody.

MTX-thyroglobulin also inhibited binding of the hybrid antibody to MTX-HSA, although not as efficiently as MTX-HSA. Thus in the second test where MTX-HSA at 5 μg ml$^{-1}$ of MTX inhibited binding of the hybrid antibody by 99%,

**Table III** Examples of simultaneous reaction of hybrid hybridoma culture supernatant fractions with tumour cells and MTX-HSA

| | *Cells incubated with*[a] | | *MLF*[b] *after reaction with FITC anti-HSA with cells of* | |
|---|---|---|---|---|
| *Purification method* | *Fraction/ antibody* | *Admixed with* | *791T* | *CoLo205* |
| A[c] | Protein A-pH 6 | MTX-HSA | 23 | 21 |
| | | – | 18 | 16 |
| | Protein A-pH 3 | MTX-HSA | 307 | 31 |
| | | – | 19 | 22 |
| B[d] | MTX-agarose-unbound | MTX-HSA | 26 | 28 |
| | | – | 23 | 26 |
| | MTX-agarose-pH 3 | MTX-HSA | 194 | 45 |
| | | – | 16 | 18 |
| – | 791T/36 | MTX-HSA | 23 | 53 |
| | | – | 15 | 21 |
| – | | MTX-HSA | 19 | 55 |
| – | | – | 15 | 19 |

[a]Cells were incubated with a mixture of antibody fractions and MTX-HSA washed and bound MTX-HSA subsequently detected with FITC labelled anti-HSA. [b]MLF = mean linear fluorescence per cell. [c]Antibody had been previously bound and eluted from MTX-agarose. [d]Antibody had been previously bound and eluted from protein A at pH 3.

**Table IV** Inhibition of binding of purified hybrid antibody to MTX-HSA

| Test no. | Conc. of hybrid antibody (µg ml⁻¹) | Inhibitor: Material | Inhibitor: Conc. (µg ml⁻¹)[a] | Reduction in ELISA absorbence (%) |
|---|---|---|---|---|
| 1 | 100 | MTX-HSA | 1 | 88 |
| | | | 5 | 99 |
| | | | 20 | 93 |
| | | MTX | 10 | 54 |
| | | | 20 | 67 |
| | | | 50 | 80 |
| | | | 100 | 89 |
| | | HSA | 10 | 13 |
| | | | 20 | 14 |
| | | | 50 | 25 |
| | | | 100 | 37 |
| 2 | 100 | MTX-HSA | 1 | 76 |
| | | | 5 | 99 |
| | | | 20 | 99 |
| | | MTX-thyroglobulin | 5 | 49 |
| | | | 10 | 65 |
| 3 | 100 | MTX-HSA | 1 | 88 |
| | | | 5 | 99 |
| | | | 10 | 99 |
| | | MTX-thyroglobulin | 1 | 55 |
| | | | 5 | 40 |
| | | | 10 | 57 |
| 4 | 6 | MTX-HSA | 2.5 | 97 |
| | | | 5 | 99 |
| | | | 10 | 99 |
| | | MTX | 2.5 | 25 |
| | | | 5 | 24 |
| | | | 10 | 42 |
| | | | 100 | 70 |
| | | HSA | 2.5 | 1 |
| | | | 5 | 2 |
| | | | 10 | 0 |
| | | | 20 | 0 |
| | | | 100 | 0 |

[a]Concentration in terms of MTX.

MTX-thyroglobulin at the same MTX concentration inhibited by only 49%. In the third test, where MTX-thyroglobulin was again compared with MTX-HSA, 1 µg ml⁻¹ of MTX as MTX-thyroglobulin inhibited binding by 55%, compared with 88% with MTX-HSA. In the final test, the concentration of antibody in the ELISA assay was decreased to 6 µg ml⁻¹ to increase the sensitivity of detection of inhibition. MTX as MTX-HSA produced virtually total inhibition of binding at 2.5 µg ml⁻¹ and upwards, whereas free MTX at 2.5 µg ml⁻¹ reduced binding by only 25%. Increasing the concentration of the free drug up to 100 µg ml⁻¹ reduced inhibition of hybrid binding to 70%. In this test HSA alone, at concentrations equivalent to MTX-HSA conjugate at up to 100 µg ml⁻¹ of MTX did not inhibit the binding of the hybrid antibody.

### *Cytotoxicity of MTX and MTX-HSA in combination with monoclonal antibodies*

Osteogenic sarcoma 791T and bladder carcinoma T24 cells were treated *in vitro* with the bispecific antibody fraction at a constant concentration of 3 µg ml⁻¹, and free MTX, titrated from 100 ng ml⁻¹ to 1 ng ml⁻¹. As expected from the binding characteristics of the anti-methotrexate moiety, the bispecific antibody did not affect the cytotoxicity of free MTX: 50% inhibitory concentrations ($IC_{50}$) of 3.5 and 3.7 ng ml⁻¹ MTX were observed for 791T in the presence and absence of antibody, respectively, and 1.9 and 1.7 ng ml⁻¹ in the case of T24.

An initial experiment was then performed with MTX-HSA in which the bispecific antibody was again used at 3 µg ml⁻¹ (Figure 1). The MTX-HSA conjugate alone was slightly more active against T24 than 791T, reflecting the greater sensitivity of T24 to the free drug. In the case of T24 cytotoxicity was virtually the same whether or not antibody was present, but the antibody signficantly enhanced the effect against 791T cells at concentrations of conjugate below 3 µg ml⁻¹ in terms of MTX content.

**Figure 1** Titration of MTX-HSA conjugate against osteogenic sarcoma 791T and bladder carcinoma T24 cells with or without anti-gp72/anti-MTX bispecific antibody (3 µg ml⁻¹). (□) 791T cells plus antibody, (▲) 791T cells minus antibody, (■) T24 cells plus antibody, (○) T24 cells minus antibody. The effect of MTX-HSA against 791T is enhanced at concentrations in the range equivalent to 0.1–3 µg ml⁻¹ MTX in the presence of antibody ($P<0.01$ to $P<0.001$). Cytotoxicity of MTX-HSA is greater for T24 than 791T cells, but is not augmented by the bispecific antibody.

The lack of augmentation at higher conjugate concentrations suggested that the system might be in antigen excess, so further tests on 791T were conducted with MTX-HSA at a low (non-toxic) dose of 0.25 µg ml⁻¹, and antibody was titrated downwards from 50 µg ml⁻¹ (Figure 2). The cytotoxicity of MTX-HSA was markedly increased by addition of bispecific antibody, the response correlating with antibody concentration. In contrast, the monospecific anti-MTX antibody fraction and parental 791T/36 (anti-gp72) did not induce augmentation of cytotoxicity by MTX-HSA, and addition of bispecific antibody alone (i.e. without the conjugate) did not affect target cell survival.

In order to evaluate the specificity of the effect of the bispecific antibody, experiments were also carried out with osteogenic sarcoma 788T, which, like 791T, expresses a high concentration of cell surface gp72 sites (about $5 \times 10^5$ per

**Figure 2** Titration of anti-gp72/anti-MTX bispecific antibody and monospecific anti-gp72 and anti-MTX against 791T cells together with MTX-HSA (equivalent to 0.25 µg ml⁻¹ MTX). (□) Bispecific antibody plus MTX-HSA, (▲) anti-MTX plus MTX-HSA, (■) anti-gp72 plus MTX-HSA, (○) bispecific antibody alone. The bispecific antibody promotes significant cytotoxicity in a concentration-dependent manner against 791T by MTX-HSA compared with MTX-HSA alone which is inactive at 0.25 µg ml⁻¹ MTX (*$P<0.01$, **$P<0.001$). The monospecific antibodies do not significantly increase cytotoxicity above background, and the bispecific antibody is inactive in the absence of MTX-HSA.

cell), and tumour lines T24 and C170 which express only about 5–10% of this gp72 level. These lines were chosen because they have similar growth rates to 791T, and similar sensitivity to MTX. The results (Figure 3) show that 788T was highly susceptible to the combined action of MTX-HSA and bispecific antibody, but T24 and C170 were unaffected at the concentrations tested. In this experiment MTX-HSA (0.25 μg $ml^{-1}$) in the absence of antibody produced cytotoxicities of 3% against 788T and below zero against T24 and C170. The selective effect of bispecific antibody against 791T and 788T confirms that binding of the anti-gp72 moiety was necessary for the mediation of cytotoxicity.

A comparison between free MTX and MTX-HSA was carried out using the 788T cell line and bispecific antibody at a constant concentration of 10 μg $ml^{-1}$, which was highly effective with this cell line (Figure 3). As shown in Figure 4, the antibody produced no change in the titration of free drug, but again enhanced the effect of MTX-HSA at low conjugate concentrations, although there was no difference at the highest concentrations used. This synergism with the conjugate but not free MTX at the lower equitoxic doses correlates with greater binding affinity of the bispecific antibody for conjugated drug, and the availability of more MTX residues per mole of conjugate compared with the free drug (i.e. target cell binding of one residue of MTX in the conjugate can result in internalisation of many others on the same molecule).

### Discussion

The aim of the present study was to generate a hybrid hybridoma producing bispecific monoclonal antibody reactive with gp72 human tumour associated antigen and MTX in the form of a MTX-HSA conjugate, to isolate the antibody from hybridoma culture supernatant and to examine its effect on the cytotoxicity of MTX-HSA.

Bispecific antibodies can be made by chemical conjugation; the use of heterobifunctional reagents allows production of bispecific antibodies in high yield (Glennie et al., 1988), although preparation of appropriate antibody fragments may be a problem, as with the IgG2b 791T/36 in the current study. Alternatively, fusion of two hybridomas or a hybridoma with immune spleen cells can be undertaken, with appropriate physical (Karawajew et al., 1987) or biochemical selection of hybrid hybridomas (Webb et al., 1985; Corvalan & Smith, 1987). Once established, hybrid hybridomas can produce a continual supply of antibody, although the

**Figure 3** Selectivity of augmentation of MTX-HSA cytotoxicity by anti-gp72/anti-MTX bispecific antibody. (□) Osteogenic sarcoma 788T cells, (▲) bladder carcinoma T24 cells, (■) colon carcinoma C170 cells. MTX-HSA was used at 0.25 μg $ml^{-1}$ MTX concentration. Cytotoxicity against 788T cells which express high levels of gp72 is highly significant ($P < 0.001$ at all antibody concentrations), but T24 and C170 with low gp72 expression are unaffected.

**Figure 4** Comparative titrations of MTX and MTX-HSA against osteogenic sarcoma 788T cells with or without bispecific antibody (10 μg $ml^{-1}$). (■) MTX plus antibody, (O) MTX minus antibody, (□) MTX-HSA plus antibody, (▲) MTX-HSA minus antibody. MTX-HSA is less cytotoxic than MTX, but its effect is enhanced by bispecific antibody (*$P < 0.01$, **$P < 0.001$) at the lower conjugate concentrations. Cytotoxicity by MTX is unaffected by the antibody.

theoretical maximum yield of bispecific antibody will be 50% of the total immunoglobulin synthesised, the remainder in this case being bivalent parent antibodies. It should be appreciated that much lower proportions of bispecific antibody can be produced as a result of other heavy:light chain reassociations (Milstein & Cuello, 1984). These considerations means that it will always be necessary when using the hybrid-hybridoma technique to develop strategies for purification of the bispecific antibody from the other antibodies being produced by the hybridoma. Overall, the yield of hybrid antibody from the 516 hybrid hybridoma averaged 8 μg $ml^{-1}$ of culture supernatant, an acceptable yield for monoclonal antibodies produced by *in vitro* culture. However, the total immunoglobulin recovered was 61 μg $ml^{-1}$, with proportions of 60% anti-gp72, 26% anti-MTX and 13% bispecific, indicating that considerable non-productive association of heavy and light chains, or preferential association of homologous heavy chains, may be occurring.

The definitive demonstration that fractions from the purification procedures contained bispecific antibody and not simply a mixture of two co-purified antibodies came from the flow cytometry assays designed to detect bridging between cell surface bound antibody and MTX in the form of MTX-HSA (Table III). The fractions containing both isotypes and reactive with both antigens when tested separately, could react simultaneously with gp72 antigen on 791T cells and with MTX-HSA.

It was proposed that the bispecific antibody produced should have highest affinity for MTX conjugated to HSA. Mice were originally immunised with such a conjugate and initial screening for antibody was carried out against MTX-HSA in the continued presence of the free MTX in HMT medium to encourage selection of those antibodies with preferential binding to the conjugate. The binding of purified hybrid antibody to immobilised MTX-HSA was inhibited by soluble MTX-HSA, but not by HSA, and was poorly inhibited by an equivalent amount of free MTX (Table IV). MTX-thyroglobulin also inhibited binding of the bispecific antibody but only about 50% as efficiently as MTX as MTX-HSA. Overall the interpretation is that this bispecific antibody reacts with highest affinity against MTX conjugated to HSA, although it is reacting with the MTX moiety and not with the HSA moiety.

The higher affinity of the bispecific antibody for conjugated MTX compared to free MTX is reflected in the augmentation of cytotoxicity by MTX-HSA but not the free drug *in vitro* (Figure 4). An additional factor is the ability of target cells to internalise up to 40 MTX molecules on each molecule of MTX-HSA cross-linked by one MTX residue to a gp72 antigen site, compared with only a single MTX molecule in the case of free drug.

Using osteogenic sarcoma 788T as the target cell line, 70% cytotoxicity was achieved using an antibody concentration of 1.6 $\mu g\ ml^{-1}$ and MTX-HSA at a concentration equivalent to 0.25 $\mu g\ ml^{-1}$ MTX (Figure 3), representing a molar ratio (MTX:Ig) of about 50:1. Previous studies have shown that conjugates of anti-gp72 antibody coupled directly to MTX-HSA by a carbodiimide reaction give similar activity against this cell line at molar ratios around 30:1 (Garnett *et al.*, 1983). Thus, the bispecific antibody was almost as efficient as a targeting vector as the chemically linked monospecific parental antibody. However, saturation of osteosarcoma 791T cells was apparently not reached by bispecific antibody even at concentrations up to 50 $\mu g\ ml^{-1}$ (Figure 2), indicating low affinity or immunoreactivity of the purified fraction. If this could be improved, the targeting efficiency might equal or exceed that of chemically coupled anti-gp72 antibody.

Other studies using bispecific antibodies recognising plant toxins have also shown cytotoxic activities similar to those obtained with conventional immunotoxin conjugate. Thus, Webb *et al.* (1985) produced a hybridoma with anti-ricin toxin A chain (RTA) and anti-prostate carcinoma activity, and showed that it could mediate selective cytotoxicity against prostate tumour cells using RTA at $10^{-9}$ M together with $NH_4Cl$ as a potentiator. Glennie *et al.* (1988) showed that chemically reconstituted bispecific antibodies recognising an immunoglobulin idiotype on guinea pig leukaemia cells and the toxin saporin could increase the toxic effect of saporin for leukaemia cells by almost 5 log units compared to the toxin alone.

Further studies with this bispecific antibody will examine *in vivo* its ability to localise in gp72 positive human tumour xenografts, to alter the biodistribution of MTX-HSA and to bring about selective targeting of this conjugate to tumours. Earlier studies with covalent conjugates of 791T/36 antibody and MTX have shown blood survival and site specific localisation of both antibody and drug moieties of the conjugates in xenografts (Pimm *et al.*, 1988) and blood survival of both moieties and tumour localisation of at least antibody in primary colo-rectal carcinoma (Ballantyne *et al.*, 1988). However, the maximum molar ratio of MTX to antibody feasible with those conjugates, at about 3:1, is viewed as a limitation to their therapeutic efficacy (Pimm *et al.*, 1988). It has been shown in the present studies that bispecific antibody can react with MTX-HSA conjugates with up to 40 MTX molecules per HSA molecule. It is therefore feasible that each bispecific antibody molecule deposited in tumour tissue could, in capturing one such molecule via only one of its MTX groups, encourage selective localisation of many more drug molecules than is possible with direct covalent conjugation of the drug to 791T/36 monoclonal antibody.

This work was supported by the Cancer Research Campaign, London, UK. We thank Mr O.F.H. Roberts for carrying out flow cytofluorimetry, and Ms D.S. Betts, Ms W. Griffiths and Mr P. Butler for their assistance.

### References

AUSTIN, E.B., ROBINS, R.A., DURRANT, L.G., PRICE, M.R. & BALDWIN, R.W. (1990). Human monoclonal anti-idiotypic antibody to tumour associated antibody 791T/36. *Immunology* (in the press).

BALDWIN, R.W., BYERS, V.S. & PIMM, M.W. (1988). Monoclonal antibodies and immunoconjugates for cancer treatment. In *Cancer Chemotherapy and Biological Response*, Pinedo, H.M., Longo, D.L. & Chabner, B.A. (eds) p. 397. Elsevier Science Publishers: Amsterdam.

BALLANTYNE, K.C., PERKINS, A.C., PIMM, M.V. & 5 others (1988). Biodistribution of a monoclonal antibody-methrotrexate (791T/36-MTX) in patients with colorectal cancer. *Int. J. Cancer*, Suppl. 2, 103.

CORVALAN, J.R.F., SMITH, W. GORE, V.A., BRANDON, D.R. & RYDE, P.J. (1987*a*). Increased therapeutic effect of vinca alkaloids targeted to tumour by a hybrid-hybrid monoclonal antibody. *Cancer Immunol. Immunother.*, **24**, 138.

CORVALAN, J.R.F., SMITH, W., GORE, C.A. & BRANDON, D.R. (1987*b*). Specific *in vitro* and *in vivo* drug localisation to tumour cells using a hybrid-hybrid monoclonal antibody. *Cancer Immunol. Immunother.*, **24**, 133.

CORVALAN, J.R.F. & SMITH, W. (1987). Construction and characterisation of a hybrid-hybrid monoclonal antibody recognising both carcinoembryonic antigen (CEA) and vinca alkaloids. *Cancer Immunol. Immunother.*, **24**, 127.

EMBLETON, M.J., GUNN, B., BYERS, V.S. & BALDWIN, R.W. (1981). Antitumour reactions of monoclonal antibody against a human osteogenic sarcoma cell line. *Br. J. Cancer*, **43**, 582.

GARNETT, M.C., EMBLETON, M.J., JACOBS, E. & BALDWIN, R.W. (1983). Preparation and properties of a drug-carrier-antibody conjugate showing selective antibody directed cytotoxicity *in vitro*. *Int. J. Cancer*, **31**, 661.

GLENNIE, M.J., BRENNAND, D.M., BRYDEN, F. & 4 others (1988). Bispecific $F(AB')_2$ antibody for the delivery of saporin in the treatment of lymphoma. *J. Immunol.*, **141**, 3663.

KARAWAJEW, L., MICHEEL, B., BEHRSING, O. & GAESTEL, M. (1987). Bispecific antibody-producing hybrid hybridomas selected by a fluorescence activated cell sorter. *J. Immunol. Meth.*, **96**, 265.

MILSTEIN, C. & CUELLO, A.C. (1984). Hybrid hybridomas and the production of bi-specific monoclonal antibodies. *Immunol. Today*, **5**, 299.

PIMM, M.V., CLEGG, J.A., GARNETT, M.C. & BALDWIN, R.W. (1988). Biodistribution and tumour localization of a methotrexate-monoclonal antibody 791T/36 conjugate in nude mice with human tumour xenografts. *Int. J. Cancer*, **41**, 886.

ROE, R., ROBINS, R.A., LAXTON, R.R. & BALDWIN, R.W. (1985). Kinetics of divalent monoclonal anitbody binding to tumour cell surface antigens using flow cytometry: standardization and mathematical analysis. *Molec. Immunol.*, **22**, 11.

WEBB, K.S., WARE, J.L., PARKS, S.F., WALTHER, P.J. & PAULSON, D.F. (1985). Evidence for a novel hybrid immunotoxin recognizing A-chain by one antigen-combining site and a prostate-restricted antigen by the remaining antigen-combining site: potential for immunotherapy. *Cancer Treat. Rep.*, **69**, 663.